# The Issue of Gender Bias Represented in Authorship in the Fields of Exercise and Rehabilitation: A 5-Year Research in Indexed Journals

**DOI:** 10.3390/jfmk8010018

**Published:** 2023-01-30

**Authors:** Natascia Rinaldo, Giovanni Piva, Suzanne Ryder, Anna Crepaldi, Alba Pasini, Lorenzo Caruso, Roberto Manfredini, Sofia Straudi, Fabio Manfredini, Nicola Lamberti

**Affiliations:** 1 Department of Neuroscience and Rehabilitation, University of Ferrara, 44121 Ferrara, Italy; 2 PhD Program in Environmental Sustainability and Wellbeing, Department of Humanities, University of Ferrara, 44121 Ferrara, Italy; 3 Instituto Maimonides de Investigation Biomedica, 14005 Cordoba, Spain; 4 Department of Environment and Prevention Sciences, University of Ferrara, 44121 Ferrara, Italy; 5 Department of Medical Sciences, University of Ferrara, 44121 Ferrara, Italy; 6 University Center for Studies on Gender Medicine, University of Ferrara, 44121 Ferrara, Italy

**Keywords:** gender bias, authorship position, exercise therapy, journal ranking, rehabilitation, exercise, gender

## Abstract

Despite progress made in recent decades, gender bias is still present in scientific publication authorship. The underrepresentation of women and overrepresentation of men has already been reported in the medical fields but little is known in the fields of exercise sciences and rehabilitation. This study examines trends in authorship by gender in this field in the last 5 years. All randomized controlled trials published in indexed journals from April 2017 to March 2022 through the widely inclusive Medline dataset using the MeSH term “exercise therapy” were collected, and the gender of the first and last authors was identified through names, pronouns and photographs. Year of publication, country of affiliation of the first author, and ranking of the journal were also collected. A chi-squared test for trends and logistic regression models were performed to analyze the odds of a woman being a first or last author. The analysis was performed on a total of 5259 articles. Overall, 47% had a woman as the first author and 33% had a woman as the last author, with a similar trend over five years. The trend in women’s authorship varied by geographical area, with the higher representation of women authors in Oceania (first: 53.1%; last: 38.8%), North-Central America (first: 45.3%; last: 37.2%), and Europe (first: 47.2%; last: 33.3%). The logistic regression models (*p* < 0.001) indicated that women have lower odds of being authors in prominent authorship positions in higher-ranked journals. In conclusion, over the last five years, in the field of exercise and rehabilitation research, women and men are almost equally represented as first authors, in contrast with other medical areas. However, gender bias, unfavoring women, still exists, especially in the last authorship position, regardless of geographical area and journal ranking.

## 1. Introduction

Gender disparity in academia, specifically in academic publishing, remains a persistent problem, despite the progress made in the last two decades [1,2,3]. Although many initiatives have been undertaken to shrink the bias (e.g., new policies and regulations, and initiatives taken by individual journals to improve equity, transparency, diversity, and inclusion in the publishing process), and despite the high prevalence of women as undergraduate, graduate, and PhD students in many countries and fields [4], women reach higher positions in academia in lower numbers than men [2,5,6,7] and at a slower rate than men [8,9]. Moreover, several studies have shown gender disparities in earnings [10], funding [11,12,13], and patenting [14], even though they are slowly decreasing [2,3,15]. However, this disparity has increased during and after the COVID-19 pandemic [16].

A straightforward way to quantify the presence of women in science is to analyze the academic output in bibliometric fields and specifically the authorship positions, as it often mirrors the hierarchical structure of academia. First and last author positions can be used as a proxy of the academic career/success of a person [17]. The first authors are usually early-career researchers (such as PhD students or postdocs), whereas the last authors are mostly senior researchers and/or principal investigators [18,19]. This is true for several fields of science, including but not limited to life science, chemistry, medicine, and others [20,21,22]. Consequently, the first and last authors have more significant reputations in comparison to the other coauthors [17,20]. Moreover, the scientific productivity of a researcher is an important factor in career progression [23]; therefore, gender inequalities in academic publishing could be among the causes of the underrepresentation of women and the overrepresentation of men, in high academic positions [24].

On average, men publish more papers than women [15], but with different proportions based on the academic fields and subfields [1], with women generally publishing fewer papers in more prestigious fields (e.g., global health, neuroscience, psychology, medicine) [25]. Moreover, men tend to predominate in the most prestigious author positions [15], becoming more pronounced particularly when looking at the last positions [26,27,28,29].

The general underrepresentation of women in academic publishing is particularly evident in the medical field [24,30,31], although it has seen an increased proportion of women in recent decades [32,33,34]. Filardo et al. [35] reported an increase from 27% to 34% of women as first authors between 1994 and 2014 in the medical research. However, the number of women was lower than that of men in all of the considered years, and it seemed to have reached a plateau or even declined in some journals [35]. Despite the apparent decrease in the gender gap, gender inequalities persist, especially when looking at senior positions. West et al. [15] analyzed all of the articles published in the JSTOR corpus between 1990 and 2011 (4.2 millions published papers), and reported that while there have been important gains in parity in the first author position, the proportion of women as last authors and in the overall authorship remains disproportionately low, regardless of the research area [15]. Another important source of bias is the geographical area in which the study was conducted. The majority of studies come from North America and Europe and Oceania, whereas Africa and Asia are underrepresented [1,35].

Despite the number of studies focused on gender representation in several medical fields, only a few of them have analyzed the research area of sport and exercise science [36,37]. Dynako et al. [36], analyzing the proportion of women in two US sports medicine journals over 30 years (from 1985 to 2016), reported a very low percentage of women in the first and corresponding author position, although it was generally increasing over time. Recently, Martinez-Rosalez et al. [37] analyzed gender in the leading authorship positions of randomized controlled trials (RCTs) published from 2000 to 2020 in 14 sports science journals. They underlined a significant gap between women and men in the first and last authorship, with women representing a quarter of the former and a fifth of the latter positions, albeit with an increase of 0.5% points per year [37].

However, to our knowledge, the majority of studies on gender bias are focused on a limited number of journals, usually those in the top ranking [38], and no research has considered all the papers published in the area of sports science and exercise in recent years.

The main aim of this study is to examine the gender inequality in prominent authorship positions in all RCTs in the field of exercise, sports and rehabilitation research published in any scientific indexed journal during the last 5 years (from 2017 to 2022), highlighting the possible influence of geographic area and the ranking of the journals.

## 2. Materials and Methods

We collected all the RCT papers that were published from 1 April 2017 to 31 March 2022 in the Medline dataset. The search strategy included the medical subjects headings (MeSH) term “exercise therapy”, which encompasses a wide range of studies from sports medicine to rehabilitation. We then applied the PubMed filter for “randomized controlled trial” to obtain the final sample of manuscripts.

The identification of the binary gender of the first and the last authors was done by F.M., G.P., S.Z. and N.L. through photographs and gender pronouns on the authors’ profiles on ResearchGate and other platforms (i.e., Google Scholar, institutional profiles, personal profiles, or social media). When the gender of either the first or the last author could not be identified, the article was excluded from further analyses.

### 2.1. Variables of Interest

For each article, data were collected on the year of publication, journal, country of affiliation of the first author, and ranking of the journal. For authors with multiple institutional affiliations, we chose the one that corresponded to the actual position or the institution where the authors spent most of their time. The countries were then grouped into continents (Africa, Asia, Europe, North and Central America, South America, and Oceania) for statistical analysis purposes. The journal ranking was represented by quartiles based on the journal impact factor. This was obtained from the website Clarivariate Analytics Journal Citation Reports by Web of Science, and we chose the journal quartile of the years of publication. The journals not indexed in the Web of Science database were arbitrarily categorized in the fourth quartile.

### 2.2. Statistical Analysis

Descriptive statistics are reported as absolute and relative frequencies. We reported the percentage of gender authorship stratified by years of publication, continent of origin of the first author, and ranking of the journal. Comparisons between groups were performed using a Chi-square test for trends (when applicable).

To analyze the odds of being a woman in one of the two leading positions, we performed two multiple logistic regression models. For the first model, we used as the dependent variable the gender of the first author. In the second model, the gender of the last author was the dependent variable. As independent variables, we inserted the following for both models: time of publication (as a continuous incremental variable, to avoid the bias of categorization); continent of origin of the first author (insert as a categorical variable; Europe as the reference continent); and ranking of the journal (expressed as the quartile of the impact factor of each journal, inserted as a categorical variable; the first quartile as the reference quartile).

## 3. Results

**Figure 1:**
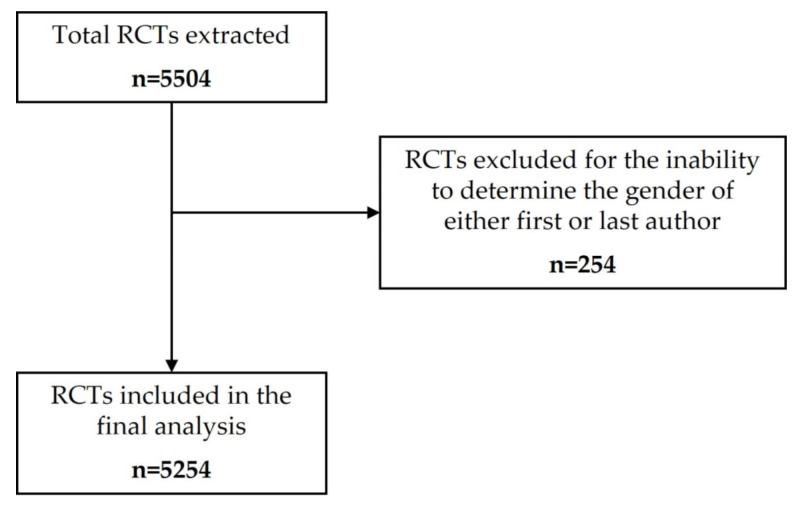
Flowchart of the study.

A total of 5504 articles were extracted. Due to the inability to determine the gender of either the first or the last author, 254 (4.6%) papers were excluded from the final analysis, which was conducted on 5259 articles (Figure 1).

### 3.1. Gender Bias over Time

**Figure 2:**
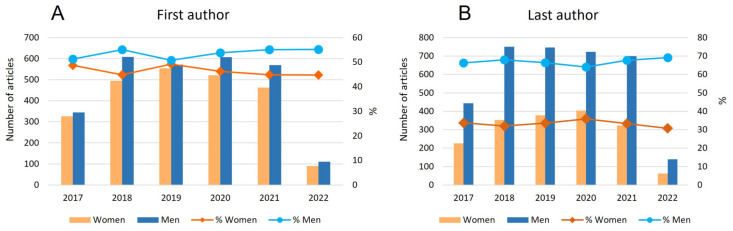
Absolute and relative frequencies of women and men as first (**A**) and last (**B**) authors from April 2017 to March 2022.

Table 1 reports the frequency of gender authorship overall and by year of publication. In total, across all years, journals, and geographical areas, 2449 (46.6%) articles had a woman as the first author, 1756 (33.4%) had a woman as the last author, and 1105 (21%) articles had women as both first and last authors (compared to 41% with men as both first and last authors). The distribution of authorship by women remains constant in the considered years, with a consistently higher percentage of men as authors (Figure 2). Analyzing the two main positions, overall, the percentage of women as first authors is almost fifty percent, with a gender ratio close to one. The results are similar over the considered years (*p* = 0.244; ꭓ^2^ test for trend), although the percentage of women is never higher than 50%, with a variability that ranges between 49.2% and 46.2% (Table 1 and Figure 2A).

Considering the last authorship position, women published approximately one-third of the articles each year (ranging from 35.9% in 2020 to 32.0% in 2018) (Figure 2B) and the difference between the considered years is not statistically significant (*p* = 0.995; ꭓ^2^ test for trend). When we analyze the percentage change between 2017 and 2021 and 2017 and 2022, they decrease for women and increase for men for all the positions of authorship considered, even if the trend is not linear (Table 1).

### 3.2. Gender Bias by Geographical Area

The majority of the overall publications, regardless of gender, are from Europe, with more than 2000 published papers. The other two continents with high numbers of RCTs focused on exercise sciences are North-Central America and Asia (approximately 1000 in each continent). The fewest number of publications are found in Oceania and Africa, which published fewer than one hundred articles over the time period in question.

**Figure 3:**
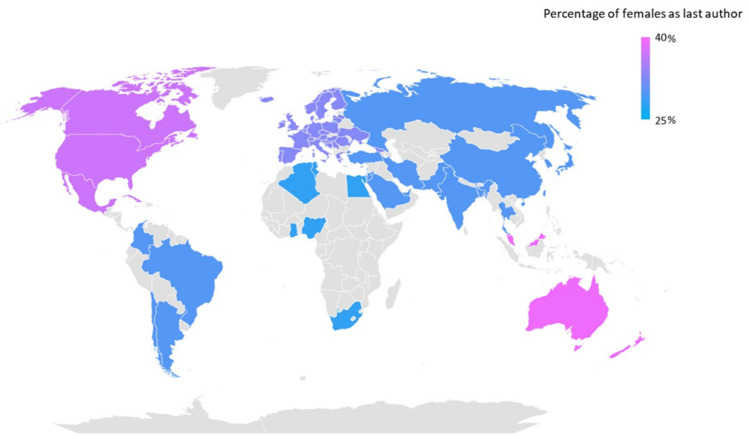
World map representing the percentage of women as last authors across the continents. Blue denotes the lower representation of women, and pink indicates the higher representation of women.

Men dominate scientific production on nearly every continent, with an underrepresentation of women both as first and last authors (Table 2). Only in Oceania are there a majority of women as first authors (53%). The first authorship is almost equally distributed among the genders in Europe (47%) and North-Central America (48%). On the other hand, in no continent is the percentage of women last authors higher than that of men. Higher values are found in Oceania (39%) and North-Central America (37%). However, it must be noted that the results found in Oceania could be biased by the lower number of overall papers published. In South America, female representation is close to 50%, but it decreases significantly when considering last authorship position (30%). In Europe and Asia, women account for slightly more than 30% of the last authorship (Figure 3). Africa is the continent with the lowest percentage of women as first (33.0%) and last (28%) author, and it is also the continent with the lowest number of publications of RCTs on the topic of sports sciences and exercise. The comparison of gender concerning the distribution of first and last authorship on the continents is significantly different (ꭓ^2^ test) (Table 2).

Table S1 reports the number of women as first and last authors across the continents, divided by year of publication. If we considered the percentage point change between 2017 and 2021, we noticed a decrease in first authorship of women in Africa, Asia, and Europe (almost 10 percentage points), and an increase in the other continents. The change in distribution across the years was significant only for Europe (*p* = 0.018; ꭓ^2^ test for trend) and almost significant for North-Central America (*p* = 0.087; ꭓ^2^ test for trend). Moreover, in Oceania and North-Central America, the percentage of first authors as women exceeded 50% in 2021. Considering the last authorship, there is a decrease in the number of women as senior authors on all continents except for Oceania (Table 1 and Figure S1); however, the changes across the years are not significant (ꭓ^2^ test for trend) (Table S1).

### 3.3. Gender Bias by Journal Ranking

Table 3 reports the differences in the frequency of articles published by men and women as either first or last author divided into quartiles based on the impact factor of the journals. The differences in publication rates between men and women are not significant (ꭓ^2^ test). Both men and women have more than 40% of their papers published in the highest quartile, followed linearly by the other quartiles (Table 3). Across the years, the results are similar, and in no year was the difference between men and women significant (ꭓ^2^ test) (Figure S1). Moreover, women had an increase in percentage from 2017 to 2021/2022 in papers published in the first, third, and last quartiles and an almost 10% decrease in the articles published in the second quartile (*p* = 0.023; ꭓ^2^ test for trend) (Table S2).

### 3.4. Multivariate Logistic Regression Models

**Figure 4:**
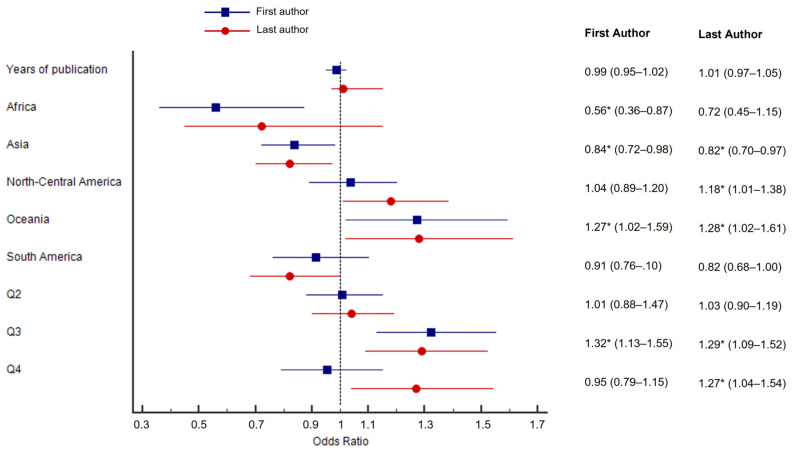
Forest plot representing the odds of being a female first (blue squares) or last (red dots) author. Values represent the odds ratio for females of being first or last author with respective 95% CIs. Asterisks represent significant odds according to multiple logistic regression models.

The results of the logistic regression models are shown in Figure 4. Both models were significant (*p* < 0.001). The odds of a woman publishing as the first author did not differ across the considered years, but the odds of being the first author in Europe are significantly higher than being the first author in Africa (the odds nearly doubled) or Asia. The greatest odds are in Oceania rather than in the other continents. Adjusting for the covariates, the odds did not differ between the first, second, and last quartile, but the odds of being a first author if you are a woman are higher if the article is published in the third quartile (Figure 4). Regarding the probability of being in the last position of authorship if you are a woman, the odds increase in North-Central America and Oceania in comparison to Europe but decrease in Asia. Moreover, the odds of being the last author of a paper published in the two bottom quartiles are approximately 1.3 points greater than the odds of being the last author of a paper published in the first quartile. Additionally, in this case, the odds of being a woman who is the last author did not change as the years progressed (Figure 4).

## 4. Discussion

This study aimed to assess the gender gap in academic publishing in the field of exercise sciences and rehabilitation. For this purpose, we collected all the RCTs published from April 2017 to March 2022 using the MeSH term “exercise therapy” focusing on the possible differences between women and men over time, by continents, and by journal ranking. The main findings of our research confirmed that the gender gap in authorship exists in this research area in all the considered years, especially when evaluating the last authorship position. Despite the confirmation of the general inequality in gendered publishing, the gender of the first authors is fairly evenly distributed among men and women. The greatest inequality was found in the last authorship positions, as men represented two-thirds of these positions. Therefore, these results did not endorse those of Martinez-Rosales et al. [37], as they found a significantly lower percentage of women as both first (24.8% vs. 46.6 in our study; ꭓ^2^ = 515.2, *p* < 0.001) and last (16.8% vs. 33.4 in our study; ꭓ^2^ = 356.9, *p* < 0.001) authors in RCTs published in top-ranking journals of the field of sports science [37]. Similar results were reported by Dynanko et al. [36]. The study of Martinez-Rosales et al. is the only one that analyses the gender bias of RTC studies focused on sports science covering a significant time frame (from 2000 to 2020), but they only considered the papers published in a limited number of selected journals, all of them in the top decile ranking of the topic “sports science”. Instead, our study is focused on the last 5 years but considered all the papers on RCTs published on the topic, regardless of the journal type or ranking, which could explain the differences in the results.

In general, we highlighted a larger proportion of women in leading positions (especially in the first position) than that reported in other biological, medical [30,31,33,35] and general research [1,15,38]. The higher percentage of women appearing as first authors in comparison to last authors is confirmed by several other studies [24,31,39,40]. This difference mirrors women’s representation in academic positions [3,8]; first authors are usually PhD students or postdocs, generally positions highly covered by women [2,41], whereas the last author is generally the senior scientist, the professor, or the principal investigator of the project, positions continuously more likely to be held by men [4,33,42].

As a confirmation of a negative gender bias towards women in positions of importance, Martinez-Rosales et al. [37] underlined that less than 20% of the editorial board positions of the top journals in sports sciences are occupied by women. These results have also been reported in other medical journals [34,43]. This underrepresentation of women on editorial boards could lead to a potential source of bias in the refereeing process of the academic journals; Tregenza [44] reported no apparent sexism in the revision process, but they also noted differences among journals in the acceptance rate of papers relative to gender. Murray et al. found that reviewers and editors tend to favor manuscripts from the same gender and country, with a lower acceptance rate in the case of a woman as the last author [45].

Several studies have reported an increase in the representation of women in prominent authorship positions focused on global or medical research [32,33,35]. Similar results were found in sports science studies that noted an increase over time in women’s first authorship but not in their last authorship [36,37]. Our analysis revealed a decrease from 2017 to 2021 in both leading positions, in accordance with other studies [30]. This difference can be due to the different periods examined and could be explained by the fact that the increase might have reached its plateau around 2009, as suggested by Filardo et al. [35]. Moreover, considering the last five years, our analysis could be biased by the influence of the COVID-19 pandemic. A study focused on 11 biomedical journals with various rankings of the BMJ group revealed that during the COVID-19 pandemic period (2020–2021), women were less represented in the most prominent authorship positions [46]. These results are partially in line with our study, as we saw a reduction in women as first authors during the pandemic (from 49.2% in 2019 to 44.9% in 2021) but no differences in last authorship (from 33.6% in 2019 to 33.3% in 2021), and this was confirmed by other studies [47].

Two important sources of bias when analyzing the representation of women in scientific authorship are the geographical area where the study was conducted and the journal ranking in which the paper was published. The representation of women as first and last authors varies widely across continents [35]. This is confirmed by our study, which found a significantly lower percentage of women as leading authors in Africa and a higher percentage in Europe, North America, and especially Oceania. The results of South America are heterogeneous, as we noted one of the highest percentage of women in the first position but one of the lowest percentages in the last position. This result is similar to the findings of other studies [36,48,49], but while confirming the higher numbers of women authorship in Europe and North America, it did not find the same results in Oceania [50,51]. However, it must be considered that the overall number of articles published in Oceania and Africa is very low (fewer than four hundred) in comparison to those published in other continents, and this could have biased the results. The low numbers of scientific articles published by authors from low-income and middle-income countries has been reported by the literature [52,53].

The fact that in several countries few women are researchers or are in leading positions depends on the sociocultural context, as women are more represented in gender-equal countries in comparison to low and middle-income countries with a more patriarchal culture (e.g., India) [2]. Low and middle-income countries are often dominated by a patriarchal, racialized, and colonial system that influences funding distribution and research hierarchy [54]. However, the underrepresentation of women in scientific publications from low-income and middle-income countries is still mainly underexplored [55]. As an example, the paper of Vuong et al. speculates that the lower number of publications by Vietnamese women could be linked to their preference to put family first in comparison to the monetary benefits of the workplace, which may have led them to choose jobs that offer better nonmonetary benefits, such as paid leave, lower weekly hours, health insurance, and social insurance [56]. The study of Merriman et al. [51] highlighted that women affiliated with low-income countries represented 1% of the last authors of the included articles. Morgan et al. [54] reported that only 25.4% and 29.7% of authors were women from low-income and middle-income countries, respectively.

Analyzing the distribution of articles by journal ranking, the univariate analysis did not show a significant difference between men and women. However, when adjusting for the year of publication and the geographical area, we noted that women are more likely to be the first and last authors if the articles are published in a third- or fourth-quartile journal. The comparisons with other studies are biased by the fact that they usually considered only publications in journals in the top ranking [32,35,37,38]. The exclusion of the non-top-ranking journals could lead to an underrepresentation of women’s authorship, as they had a higher number of publications in lower-ranking journals, as is partially demonstrated by our study. The underrepresentation of women in high-ranking journals was observed in several studies in different fields of science [38,57], albeit with some exceptions [48].

The lower numbers of women as the last authors could be linked to several factors. It has already been proven that gender disparity exists in the highest academic positions [2,8]. For the same level of responsibility, women receive smaller and fewer research grants [58,59,60] and are therefore less likely to lead research projects. Moreover, various forms of discrimination occur in the careers of women scientists that prevent them from reaching the highest academic positions, and they face more barriers than men in publishing their research and in advancing in their academic careers [9,24,61,62,63]. This discrimination also includes family reasons, such as career breaks for maternal leave and the lower productivity of women with children given that they have to balance their career with unpaid labor [64]. This is called the “Matilda effect”, which reflects the unequal opportunity between men and women and the different recognition of the same merits [65,66]. This reality is found in many scientific disciplines. Lerchenmueller and Sorenson demonstrated that women become PIs at a 20% lower rate than men in the field of life sciences [67]. However, it must be noted that gender bias is not homogeneous among the disciplines; in fact, the major underrepresentation of women in the USA has been reported in the fields of science, technology, engineering, and mathematics, whereas male underrepresentation has been reported in health care, elementary education, and domestic sphere fields [68]. Therefore, our results might be strongly influenced by the number of women that chose this particular career and educational courses.

Regardless of the reasons, the unequal representation of women in the field of sports science could lead to limited views and approaches, which limits the pursuit of diverse, inclusive, and innovative research [8,69]. Moreover, in the current academic system, academic publishing is considered an important measure of academic productivity and is deemed necessary for the progression of an academic career [3]; therefore, measures should be adopted to understand the reasons behind this gender gap in order to help reduce it.

### Strengths and Limitations

This study has several strengths and limitations. The major strength is that to the best of our knowledge, this is the first study that analyzed all of the RCT studies published in the last five years on the topic of sport and exercise research, regardless of the journal topic or ranking. This selection allowed us to draw a more complete overview of male and female representation in academic publishing in this field of science. Moreover, we decided to manually assign gender to all the first and last authors using photographs, pronouns, and social networks, avoiding the aid of automatic software (i.e., gender.io, Gender-API.com, or Gendermetrics. NET); although time-consuming and partially subjective, this method allowed us to avoid the mistakes made by automatic tools that could be prone to errors in determining gender or when gender cannot be identified when just the initials are reported. Moreover, we identified the quartile of each journal based on its impact factor to consider the journal ranking in our analysis. The main limitation is that we did not identify the number and gender of the coauthors, thus limiting our analysis to just the two leading positions. Moreover, our analysis, as well as similar published research, used a binary construct of gender, although it must be evaluated on a spectrum. Other limitations are that we only analyzed the last five years and that we only collected RCTs, excluding all other types of publications (i.e., reviews, case studies, cross-sectional studies, and observational studies). The last limitation is the systemic bias of using Medline indexing and that we used only this dataset and only one research term (“exercise therapy”). However, given the large number of articles included in the analysis, we consider these limitations to be minimal, and we argue that this study is relevant to the knowledge on the representation of gender in leading and senior authorship positions in academic publishing.

## 5. Conclusions

In conclusion, this study provided an overview of the representation of gender in relation to first and last authorship positions in the field of exercise sciences and rehabilitation. Our study confirms a general underrepresentation of women, although different perspectives offer varying results. The proportion of women as first and last authors was found to be higher than that reported by other studies. In particular, women and men were almost equally represented as first authors, but women only represent approximately one-third of the last authors, regardless of the year of publication, geographic area, or journal ranking. This proportion decreased over time. Our multivariate analysis also showed that women are more represented in the leading positions in the lower journal rankings, unlike men. Further research is needed to expand the time frame considered and to understand the reasons behind this gender gap, which could reflect a bias in the academic hierarchy.

## Figures and Tables

**Table 1:** Description of gender authorship (first, last, both first and last, and either first or last) overall and by year of publication.

	Overall N (%)	2017 ° N (%)	2018 N (%)	2019 N (%)	2020 N (%)	2021 N (%)	2022 ^¥^ N (%)	Change from 2017 to 2021	Change from 2017 to 2022	*p* Value ^a^
No. of publications	5259	670	1103	1125	1128	1032	201	-	-	-
Woman first author	2449 (46.6)	326 (48.7)	495 (44.9)	554 (49.2)	521 (46.2)	463 (44.9)	90 (44.8)	−3.8	−3.9	0.244
Man first author	2810 (53.4)	344 (51.3)	608 (55.1)	571 (50.8)	607 (53.8)	569 (55.1)	111 (55.2)	3.8	3.9	
Woman last author	1757 (33.4)	226 (33.7)	353 (32.0)	378 (33.6)	405 (35.9)	323 (33.3)	62 (30.8)	−0.4	−2.9	0.995
Man last author	3502 (66.6)	444 (66.3)	750 (68.0)	747 (66.4)	723 (64.1)	699 (67.7)	139 (69.2)	0.4	2.9	
Woman first and last author	1105 (21.0)	155 (23.0)	203 (18.4)	248 (22.0)	251 (22.3)	208 (20.2)	40 (19.9)	−2.8	−3.1	0.544
Man first and last author	2158 (41.0)	273 (40.7)	458 (41.5)	441 (39.2)	453 (40.2)	444 (43.0)	89 (44.3)	2.3	3.6	
Woman first or last author	3101 (59.0)	397 (59.3)	645 (58.5)	684 (60.8)	675 (59.8)	588 (57.0)	112 (55.7)	−2.3	−3.6	0.584
Man first or last author	4154 (79.0)	515 (76.9)	900 (81.6)	877 (78.0)	877 (77.7)	824 (79.8)	161 (80.0)	2.9	3.1	

° April; ^¥^ March; ^a^ comparison between men and women performed through the ꭓ^2^ test for trend.

**Table 2:** Authorship comparison (first and last author) between females and males stratified by continent of origin of the first author.

	Females N (%)	Males N (%)	*p* Value ^b^
First author			0.001
Africa (*n* = 94)	31 (33.0)	63 (67.0)	
Asia (*n* = 1066)	462 (43.3)	604 (56.7)	
Europe (*n* = 2007)	947 (47.2)	1060 (52.8)	
North-Central America (*n* = 1087)	525 (48.3)	562 (51.7)	
Oceania (*n* = 371)	197 (53.1)	174 (46.9)	
South America (*n* = 634)	287 (45.3)	347 (54.7)	
Last author			0.001
Africa (*n* = 94)	26 (27.7)	68 (71.3)	
Asia (*n* = 1066)	324 (30.4)	742 (69.6)	
Europe (*n* = 2007)	669 (33.3)	1338 (66.7)	
North-Central America (*n* = 1087)	404 (37.2)	683 (62.8)	
Oceania (*n* = 371)	144 (38.8)	227 (61.2)	
South America (*n* = 634)	190 (30.0)	444 (70.0)	

^b^ Comparison performed using the ꭓ^2^ test between all continents.

**Table 3:** Distribution of articles published by women and men either as first or last author by journal ranking (quartiles based on journal impact factor).

Journal Ranking (Quartile)	Women (*n* = 3101)	Men (*n* = 4154)	*p* Value ^b^
	N (%)	N (%)	0.117
Q1	1329 (42.9)	1826 (44.0)	
Q2	890 (28.7)	1222 (29.4)	
Q3	539 (17.4)	634 (15.3)	
Q4	343 (11.1)	472 (11.4)	

^b^ Comparison performed using the ꭓ^2^ test.

## Data Availability

The dataset used in this article is available at: doi: 10.17632/9cbv8fwrv2.1.

## Supplementary Materials

The following supporting information can be downloaded at https://www.mdpi.com/article/10.3390/jfmk8010018/s1: Table S1: Absolute and relative frequencies of females as first and last author overall and by year of publication, stratified by continent of origin of the first author; Figure S1: Percentage of females as first (A) and last (B) for each continent and divided by the year of the publication; Table S2: Distribution of articles published by females as either first or last authors divided by journal ranking (quartiles) per years of publication.
