# Supplementary material for: The Issue of Gender Bias Represented in Authorship in the Fields of Exercise and Rehabilitation: A 5-Year Research in Indexed Journals
Source: J Funct Morphol Kinesiol. 2023 Jan 30;8(1):18. doi: 10.3390/jfmk8010018 (PMC9944464; doi:10.3390/jfmk8010018)
Supplement: Supplementary file 1 [file jfmk-08-00018-s001.zip › jfmk-2147012-supplementary.pdf]

SUPPLEMENTARY MATERIAL

Table S1. Absolute and relative frequencies of women as first and last author overall and by year of publication, stratified by continent of origin of the first author

	Total	2017°	2018	2019	2020	2021	2022¥	Change from 2017 to 2021	Change from 2017 to 2022	
**N of women as first author**	**% (n/tot)**	**% (n/tot)**	**% (n/tot)**	**% (n/tot)**	**% (n/tot)**	**% (n/tot)**	**% (n/tot)**			0.066
Africa	33.0 (31/94)	40.0 (4/10)	27.3 (3/11)	42.9 (9/21)	33.3 (8/24)	21.7 (5/23)	40.0 (2/5)	-18.3	0.0	
Asia	43.3 (462/1066)	47.3 (53/112)	39.5 (70/177)	43.6 (102/234)	47.2 (116/246)	40.8 (98/240)	40.4 (23/57)	-6.5	-6.9	
Europe	47.2 (947/2007)	51.1 (139/272)	47.3 (200/423)	47.4 (219/462)	45.8 (204/445)	41.4 (155/374)	44.8 (30/67)	-9.7	-6.3	
North-Central America	48.3 (525/1087)	47.9 (69/144)	40.7 (109/268)	53.7 (131/244)	48.0 (96/200)	52.2 (108/207)	50.0 (12/24)	4.3	2.1	
Oceania	53.1 (197/371)	47.1 (33/70)	59.0 (49/83)	50.7 (37/73)	47.7 (31/65)	58.1 (36/62)	61.1 (11/18)	11.0	14.0	
South America	45.3 (287/634)	45.2 (28/62)	45.4 (64/141)	44.1 (56/127)	44.6 (66/148)	48.4 (61/126)	40.0 (12/30)	3.2	-5.2	
**N of women as last author**	**% (n/tot)**	**% (n/tot)**	**% (n/tot)**	**% (n/tot)**	**% (n/tot)**	**% (n/tot)**	**% (n/tot)**			0.1104
Africa	27.7 (26/94)	40.0 (4/10)	9.0 (1/11)	23.8 (5/21)	29.2 (7/24)	30.4 (7/23)	40.0 (2/5)	-9.6	0.0	
Asia	30.4 (324/1066)	31.2 (35/112)	31.6 (56/177)	31.6 (74/234)	31.7 (78/246)	27.5 (66/240)	26.3 (15/57)	-3.7	4.9	
Europe	33.3 (669/2007)	32.0 (87/272)	29.6 (125/423)	29.3 (145/426)	37.5 (167/445)	33.7 (126/374)	28.3 (19/67)	1.7	-3.7	
North-Central America	37.2 (404/1087)	34.0 (49/144)	38.1 (102/268)	37.3 (91/244)	41 (82/200)	33.8 (70/207)	41.7 (10/24)	-0.2	7.7	
Oceania	38.8 (144/371)	37.1 (26/70)	39.8 (33/83)	37.0 (27/73)	32.3 (21/65)	46.8 (29/62)	44.4 (8/18)	9.7	7.3	
South America	30.0 (190/634)	40.3 (25/62)	25.5 (36/141)	28.3 (36/127)	33.8 (50/148)	24.8 (35/126)	26.7 (8/30)	-15.5	-13.6	

° April; ¥ March

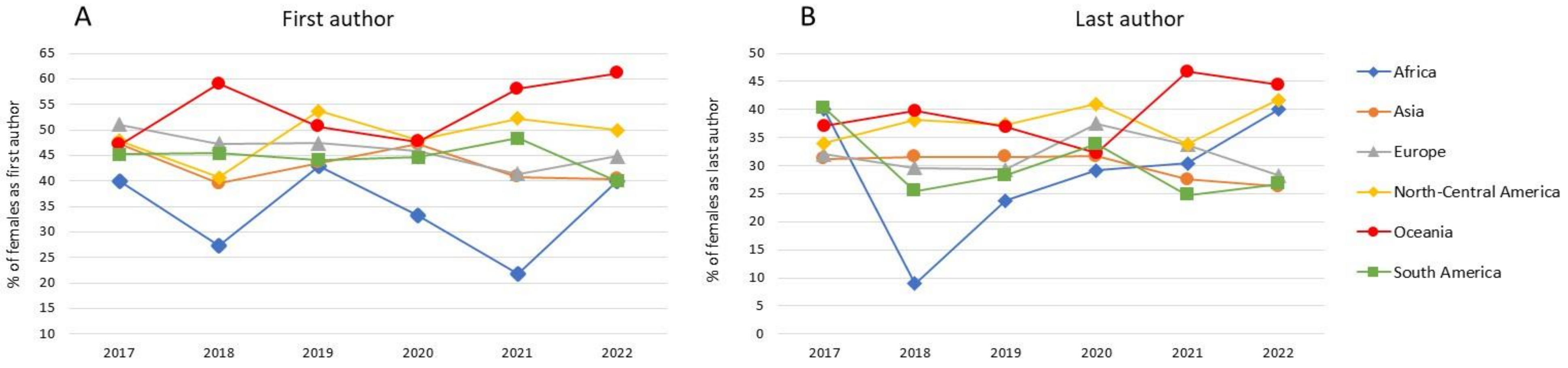

Figure S1 Percentage of women as first (A) and last (B) for each continent and divided by the year of the publication

Table S2 Distribution of articles published by women as either first or last authors divided by journal ranking (quartiles) per years of publication

**Journal ranking (percentile)**	Total	2017°	2018	2019	2020	2021	2022[¥]	Change from 2017 to 2021	Change from 2017 to 2022	P value[a]
<25	1329 (42.9)	162 (40.8)	283 (43.9)	291 (42.5)	283 (41.9)	264 (44.9)	46 (41.1)	4.1	0.3	0.023
25-49	890 (28.7)	148 (37.3)	180 (27.9)	183 (26.8)	181 (26.8)	165 (28.1)	22 (29.5)	-9.2	-7.8	
50-74	539 (17.4)	53 (13.4)	115 (17.8)	135 (19.7)	131 (19.4)	86 (14.6)	19 (17.0)	1.2	3.6	
≥75	343 (11.1)	34 (8.6)	67 (10.4)	75 (11.0)	80 (11.9)	73 (12.4)	14 (12.5)	3.8	3.9	

°April
[¥]March
[a]comparison between males and females distribution per years performed through χ^2 test for trend
